# Thermo-Responsive Smart Hydrogels: Molecular Engineering, Dynamic Cross-Linking Strategies, and Therapeutics Applications

**DOI:** 10.3390/gels12010012

**Published:** 2025-12-23

**Authors:** Jiten Yadav, Surjeet Chahal, Prashant Kumar, Chandra Kumar

**Affiliations:** 1 Department of Chemistry, University Centre for Research and Development, Chandigarh University, Mohali 140413, Punjab, India; 2 Department of Physics, University Centre for Research and Development, Chandigarh University, Mohali 140413, Punjab, India; chahalsurjeet42@gmail.com; 3 Next-Generation Magnet Development Collaboration Unit, RIKEN Center for Integrative Medical Sciences, Yokohama 230-0045, Japan; 4 Escuela de Ingeniería, Facultad de Ciencias, Ingeniería y Tecnología, Universidad Mayor, Santiago 7500994, Chile

**Keywords:** Gels, implants, drug delivery, tissue engineering, thermosensitive hydrogels

## Abstract

Temperature-responsive hydrogels are sophisticated stimuli-responsive biomaterials that undergo rapid, reversible sol–gel phase transitions in response to subtle thermal stimuli, most notably around physiological temperature. This inherent thermosensitivity enables non-invasive, precise spatiotemporal control of material properties and bioactive payload release, rendering them highly promising for advanced biomedical applications. This review critically surveys recent advances in the design, synthesis, and translational potential of thermo-responsive hydrogels, emphasizing nanoscale and hybrid architectures optimized for superior tunability and biological performance. Foundational systems remain dominated by poly(N-isopropylacrylamide) (PNIPAAm), which exhibits a sharp lower critical solution temperature near 32 °C, alongside Pluronic/Poloxamer triblock copolymers and thermosensitive cellulose derivatives. Contemporary developments increasingly exploit biohybrid and nanocomposite strategies that incorporate natural polymers such as chitosan, gelatin, or hyaluronic acid with synthetic thermo-responsive segments, yielding materials with markedly enhanced mechanical robustness, biocompatibility, and physiologically relevant transition behavior. Cross-linking methodologies—encompassing covalent chemical approaches, dynamic physical interactions, and radiation-induced polymerization are rigorously assessed for their effects on network topology, swelling/deswelling kinetics, pore structure, and degradation characteristics. Prominent applications include on-demand drug and gene delivery, injectable in situ gelling systems, three-dimensional matrices for cell encapsulation and organoid culture, tissue engineering scaffolds, self-healing wound dressings, and responsive biosensing platforms. The integration of multi-stimuli orthogonality, nanotechnology, and artificial intelligence-guided materials discovery is anticipated to deliver fully programmable, patient-specific hydrogels, establishing them as pivotal enabling technologies in precision and regenerative medicine.

## 1. Introduction

**Figure 1:**
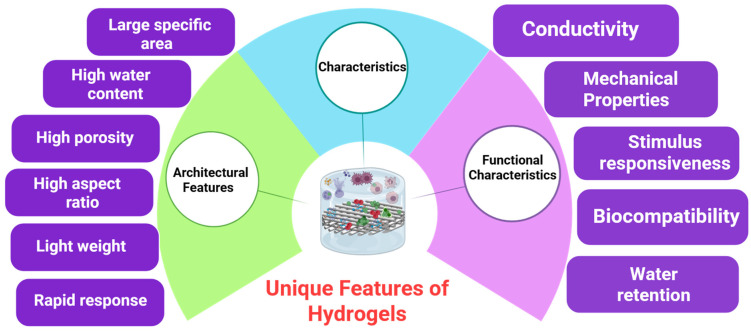
Representation of distinct characteristics of hydrogels, which are visually divided into two domains: Architectural Features and Functional Characteristics.

Hydrogels, three-dimensional (3D) webs of hydrophilic polymeric chains, have developed into a hydrogel that is a versatile material with incredible applications in all areas, such as biomedical, industrial, and agricultural engineering. Being defined by their capacity to absorb and retain large amounts of water or biological fluids, hydrogel has unusual properties due to the complexity of cross-linking of polymeric chains that give it structural stability and responsiveness to environmental stimuli [1]. The idea of hydrogel dates back to 1894, when its development into a complex type of material that is currently central to modern science and technology commenced (Figure 1). Hydrogel is an example of a materials science foundation block with extensive applications in biomedical, environmental, and industrial applications, a three-dimensional polymer network that can absorb and hold large quantities of water or biological fluids without any loss of structure [2]. These silicone-based materials recapitulate the extracellular matrix of biological tissues and are highly biocompatible, capable of condensing bioactive molecules and having tunable mechanical properties [3,4]. The historical momentum of hydrogel dates back to 1894, when Cross first reported the formation of a colloidal gel by inorganic salts, and it was the beginning of a division of study that has come to be known as the field of multidisciplinary study that involves chemistry, physics, and engineering. In the past few decades, the development of polymerization and cross-linking principles has turned hydrogels into complex, responsive systems adopting external stimuli, and this has given the hydrogel tremendous potential application in fields as diverse as wound dressings and contact lenses, agricultural soil conditioners and drug delivery vehicles [5].

**Figure 2:**
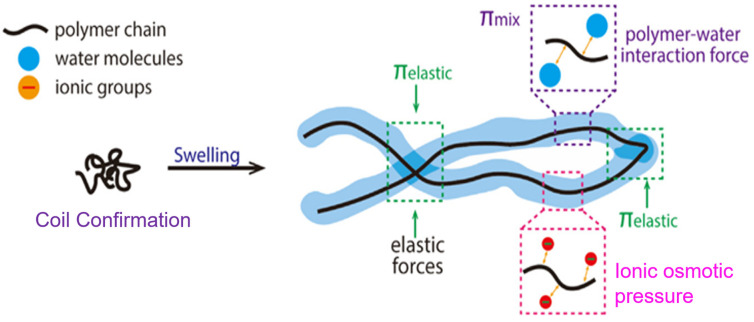
Schematic representation of the swelling process of hydrogel controlled by the interactions between the thermodynamic forces of mixing (*π*_mix_), and elastic retractive force of the polymer chains (*π*_elastic_), and ionic swelling pressure (*π*_ionic_). Reprinted with Creative Commons BY-NC-ND 4.0 [7].

However, conventional hydrogels tend to be non-dynamic in their responsiveness, and this can restrict their application in cases where the properties should be changed on demand. This has prompted the design of so-called smart or stimuli-responsive hydrogels that can accomplish reversible conformational changes in environmental cues like pH, ionic strength, light, electric fields or temperature [6]. The major peculiarity of such a materials is that hydrogels are hydrophilic, as indicated in Figure 2. The thermo-responsive hydrogel is a unique category of intelligent polymeric compounds which reveal reversible and predictable alterations in their physicochemical characteristics in reaction to temperature fluctuations, commonly within the physiological range. These types of hydrogels are usually produced by using chemical or physical cross-linking of temperature-sensitive polymers, including poly(N-isopropylacrylamide), pluronic block copolymers and naturally derived polymers with thermo-active functional groups added. The composition, cross-linking density, and architecture of the polymer can be precisely controlled to allow fine control of the lower or upper critical solution temperature, mechanical strength and swelling–deswelling properties of polymers. Due to such tunable properties, thermo-responsive hydrogels have found a very lucrative application in biomaterials and biotechnology such as controlled drug delivery, tissue-engineering scaffolds, injectable biomaterials, biosensing, and soft actuators, where the temperature-controlled response can be used to provide a spatial and temporal control over material behavior.

Smart or intelligent hydrogels are a wide category of polymeric networks that can be subjected to reversible physicochemical transitions in response to the slightest changes in the environment. Such stimuli-receptive behaviors are the result of the nature of the interaction between the molecular architecture of the hydrogel, the interaction of the polymer with the solvent, and the character of external trigger. Smart hydrogels can respond to physical stimuli (temperature, light, magnetic or electric field, pressure, ultrasound and so on), chemical stimuli (pH, ionic strength, glucose concentration, redox potential and so on), or biological stimuli (enzymes, antigens, metabolic signals and so on) [8].

**Figure 3:**
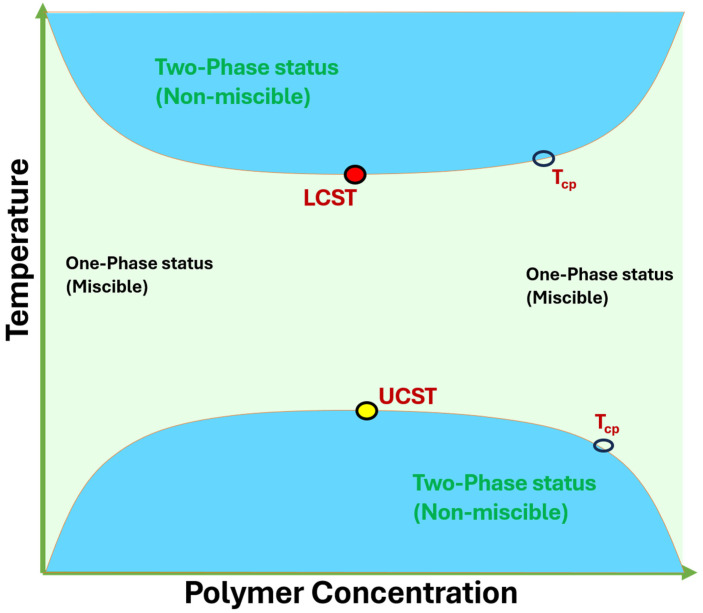
Graphical plot of LCST and UCST illustrating one phase (miscible) and two phases (non-miscible) status. Reprinted with Creative Commons CC BY 4.0 [10].

This responsiveness allows the controlled swelling–deswelling, sol–gel transitions, conformational rearrangements or degradation events to be accurately tuned by polymer composition, cross-linking density, and functional group modification. Among these more general groups, thermo-responsive hydrogels are one important subgroup in which the interactions between polymers and water are changed by temperature variations to produce phase transitions such as a lower critical solution temperature (LCST) and upper critical solution temperature (UCST) type of behavior (Figure 3). Placing thermo-responsive gels into the broader context of smart hydrogel systems, the distinctive benefits of such gels and their applications can be better put into context, especially in biomedical delivery, tissue engineering, sensing, and actuating systems [9].

Temperature-responsive nano hydrogels are designed by carefully choosing the kind of monomers, cross-linkers, and fabrication techniques to adjust the critical temperatures to biologically relevant temperatures, which are usually between 32 and 42 °C in human applications. The synthesis can be performed through the use of free radical polymerization, click chemistry, and self-assembly of amphiphilic block copolymers with nanoparticles, nanofibers, or nanocomposites often containing built-in functionalities being obtained [11]. Covalent stability is obtained by chemical cross-linking with reagents such as N, N’-methylenebisacryl amide, and reversibility is obtained by ionic cross-linking through multivalent ions. More sophisticated methods, like in situ gelling by photo-polymerization with UV or visible light initiators, allow for reducing invasiveness in the clinical environment [12,13]. Moreover, nanomaterial-based hybrid systems (such as magnetic nanoparticles (graphene oxide)) increase thermal conductivity and responsiveness and can be used to combine multimodal stimuli [14,15,16]. The characterization of these hydrogel materials is necessary to clarify the structure–property relationships [17]. Methods like differential scanning calorimetry (DSC) are used to measure enthalpies of phase transitions and critical temperatures; rheological methods measure viscoelastic properties, gelation kinetics, and shear-thinning behavior, which are important parameters in injectability. The interactions of the molecules and the density of the cross-linking of the material are determined by spectroscopic methods (Fourier-transform Infrared (FTIR) and nuclear magnetic resonance (NMR) spectroscopy. Meanwhile, scanning electron microscopy (SEM) and transmission electron microscopy (TEM) are used to present the data on the morphology of the nanoscale and porosity and swelling characteristics in detail. Under different temperatures, swelling studies measure the uptake of water that can be up to 1000 percent of dry weight, highlighting the ability of the studied hydrate [18]. One important characteristic that is required in order to be of use in biomedicine is biodegradability, which can be designed by using hydrolysable linking agents or enzymatic disinfectant sites so that it can be dissolved in a controlled manner after the function is complete.

Temperature-responsive nano hydrogels are transformative in biomedical applications [19]. To deliver drugs, they allow thermosensitive release profiles, i.e., high temperature, such as fever, external heating, or near-infrared irradiation; cause payload release out of the gel matrix; and site-specific therapy is achieved with minimized systemic toxicity. These hydrogels in tissue engineering are injectable scaffolds that gel in vivo at body temperature and enhance cartilage, bone, and neural tissue regeneration.

### Thermosensitive Polymers and Their Sol–Gel Transition Mechanism

The different classes of polymers (polysaccharide derivatives, amides, polyesters, and polyether polymers) are prepared by a combination of different approaches to polymerization, including free radical polymerization, ring-opening polymerization, chemical coupling reaction, electrostatic adherence, and double emulsion processes [20]. Their encapsulation of cells ensures viability in transplantation, protecting cells against immune action as well as allowing diffusion of nutrients. New uses are biosensing to measure temperature, wound-healing patches that can be used on irregular surfaces, and ophthalmic sustained-release formulations [21]. Outside the medical field, the materials have been applied to environmental problems, including regulated discharge of agrochemicals according to variations in soil temperature to boost crop production and reduce runoff [22].

The hydrogel network is affected by such factors as pH, ionic strength, temperature, and contact time.

**Figure 4:**
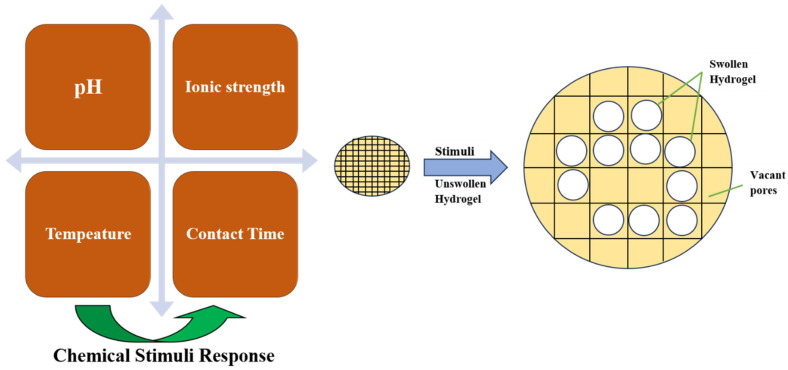
Hydrogel swelling behavior at different environmental stimuli.

Nevertheless, there remain issues, such as enhancing biocompatibility and reducing inflammatory reactions, large-scale production, and tuning response sensitization to personalized medicine [23]. Recent studies combine computer simulations, including molecular dynamics, to predict phase equilibriums and inform material design (Figure 4). Hybrids with multiple capabilities, e.g., temperature responsiveness in combination with other stimuli, will offer even more diversification, and can potentially transform areas such as oncology with hyperthermic chemotherapy.

In a broad way, this review identifies the principles of design, the physical and chemical characteristics, and biomedical uses of developed temperature-responsive nano hydrogels. Through synthesis and realization of the recent progress in synthesis strategies, mechanistic understanding of LCST/UCST dynamics and case studies in therapeutic and diagnostic innovations, this manuscript will aim to clarify the critical role of the interface between materials science and clinical demands. Conclusively, this discussion highlights the potential revolution of smart materials to address urgent medical issues and enable future progress in materials that will improve patient outcomes and sustainability.

## 2. Classification of Smart Hydrogels

Hydrogels are categorized according to their source, polymeric composition, physical structure and cross-linking type and each factor affects their biocompatibility, mechanical behavior, and stimulus responsiveness. Natural hydrogels (e.g., collagen, chitosan, alginate) offer very good biocompatibility and biodegradability but low mechanical strength, whereas synthetic hydrogels (e.g., PEG, PVA, PNIPAM) can have some mechanical and degradation characteristics that can be controlled and used in temperature-sensitive applications. The hybrid hydrogels are natural polymers with synthetic polymers to obtain both biocompatibility and construction [24].

**Figure 5:**
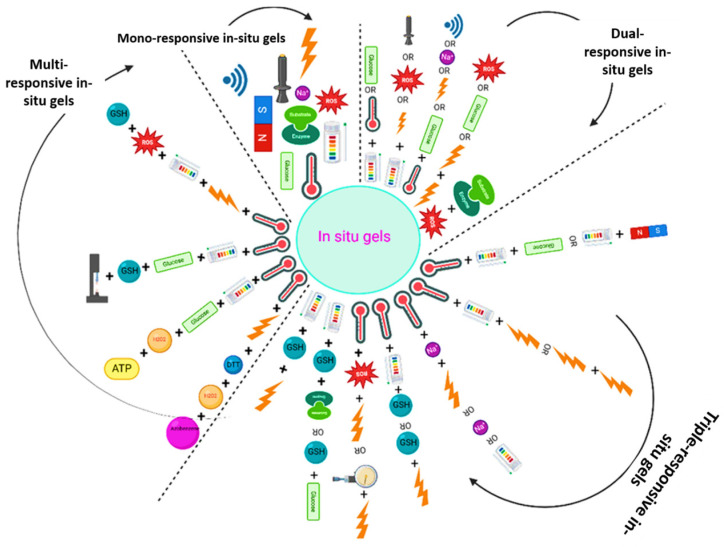
Representation of the overall stimuli-responsive in situ gel system. Reprinted with Creative Commons CC BY 4.0 [31].

Homopolymeric hydrogels are products of a monomer and exhibit clear transitions as in PNIPAM thermos-responsible gels [25]. Whereas hydrogels may be classified by their origin, polymer structure, composition, and cross-linking type, the interpenetrating polymer networks (IPNs) offer greater strength and remain sensitive to external stimuli [26]. However, these basic classifications directly affect the capacity of hydrogels to respond to external stimuli. How well natural polymers display stimuli-responsive behaviors (temperature, pH, light, or magnetic sensitivity) depends on the biocompatibility of the polymers, the tunability of the synthetic systems and the synergistic properties of hybrid hydrogels. Similarly, the molecular structure, be it a homopolymer, copolymer or an IPN, is an important parameter in determining the acuity and reversibility of such responses [27]. The stability and dynamical adaptability needed in stimuli-induced phase transitions are further dictated by physical, chemical or dual cross-linking. This knowledge of these basic classifications, as such, will present a requisite guide in the examination of advanced smart hydrogels [28]. Expanding on these structural and composition characteristics, the following section dwells on the stimuli-responsive [29,30] classification of hydrogels and the way various external signals can regulate hydrogel behavior to achieve given biomedical and environmental stimuli (Figure 5) [31].

**Figure 6:**
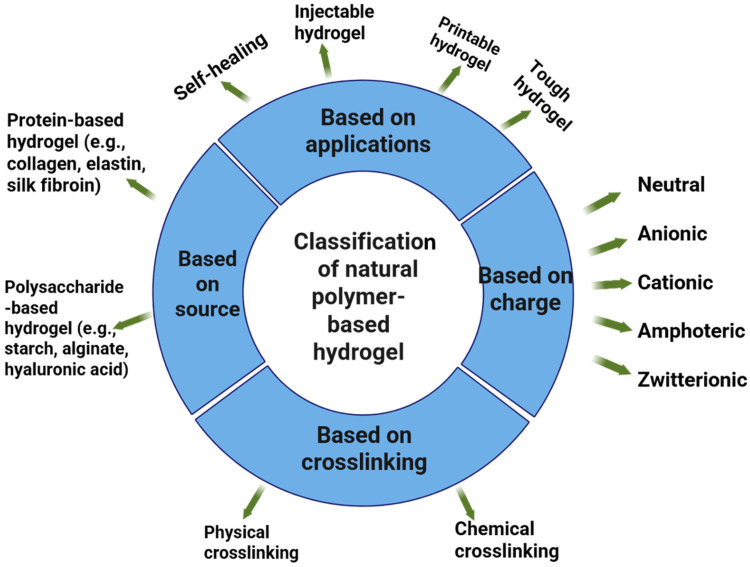
Classification of natural polymer-based hydrogels categorized by their source, charge, cross-linking mechanism, and applications. Hydrogels can be derived from proteins (e.g., collagen, elastin, silk fibroin) or polysaccharides.

The major parameters of categorizing natural polymer-based hydrogels into key classification parameters that also serve as the basis to create stimuli-responsive smart hydrogels that are dependent on specific environmental triggers and advanced applications are shown in Figure 6. Structurally, high-swelling [32], low-strength amorphous hydrogels [33] and semicrystalline hydrogels [34] provide better mechanical stability for scaffolds [28], and a supramolecular hydrogel formed through non-covalent interactions [35,36] provides dynamic, self-healing and adaptive behavior [37,38].

Alongside the usage of cross-linking to make the transition reversible with physical hydrogels [39], long-term stability with chemical hydrogels [40] and the combination of durability and responsiveness with dual cross-linked systems make it possible to apply these materials in higher biomedical systems like controlled drug delivery and tissue engineering [41].

### 2.1. Classification Based on Stimuli-Responsiveness

Hydrogels may be engineered such that they react to external stimuli, and hence, they are intelligent biomedical materials.

**Figure 7:**
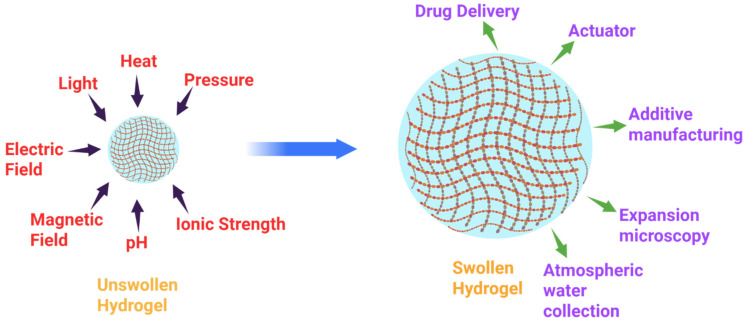
Illustration of the swelling of hydrogels upon the action of external environmental stimuli. Reprinted with Creative Commons BY-NC-ND 4.0 [7].

**Temperature-Responsive Hydrogels**: These are hydrogels that experience a phase transition upon temperature changes, which can be described by an LCST or upper critical solution temperature (UCST). The temperature-responsive hydrogels that are the most studied are PNIPAM-based nano hydrogels, which shrink above their LCST (~32 °C) because of the hydrophobic interactions. This property is utilized in drug release; the drug is released in case of a collapse of the hydrogel net due to temperature conditions (Figure 7).

**pH-Responsive Hydrogels**: Hydrogels are pH-responsive, usually because of ionizable groups in the polymer backbone. By incorporating pH and temperature sensing in nano hydrogels, nano hydrogels can be used in targeted drug delivery, including the acidic microenvironment of tumors [42].

**Multi-Responsive Hydrogels**: These are responsive to more than one stimulus, i.e., temperature, pH, light, or magnetic fields. Multi-responsive nano hydrogels are extremely flexible, and it is possible to control drug release or tissue regeneration in sophisticated physiological conditions in a very precise fashion.

**Effect of Solvent:** The impact of solvent properties was determined to play a key role in determining physicochemical behavior, injectability, diffusion dynamics and the biocompatibility of the in situ forming microparticle (ISM) system. It is clear that the density, viscosity, surface tension, and pH of solvents such as N-methyl pyrrolidone (NMP), 2-pyrrolidone (PYR), dimethyl sulfoxide (DMSO), triacetin, and glycofurol vary significantly and that these parameters together determine the nature of solvent–polymer interactions and phase inversion behavior. Solvents that were water-miscible and of low viscosity such as DMSO and NMP facilitated rapid wetting, strong dye diffusion, and high injectability, so they can be considered suitable for the formation of the internal phase of ISM systems. On the other hand, the camellia and olive oils were highly viscous, and therefore, had a long flow time, low wettability, and low diffusion, and this makes them suitable as external oil phases that can be used to control the burst release. The biological tests also validated that NMP and PYR have a better antimicrobial effect, whereas DMSO has the lowest evidence of cytotoxicity of the used solvents, which confirms its considerations in parenteral use [43]. The solvent environment is playing a predominant role in determining the thermo-responsiveness of polymers in non-aqueous media, which is mainly through controlling polymer–solvent interactions, hydrogen-bonding dynamics and through solvophilic/solvophobic balances. As shown in alcohols, including methanol, ethanol, 1-propanol and 2-propanol, a small change in the solvent polarity, hydrogen-bonding length and the ability to form sol–gel reveals significant changes in the LCST/UCST transition temperatures, sol–gel and assembly. The fact that even structurally related solvents may trigger different aggregation or dissolution processes (e.g., hydrogen bond switching of alcohols, crystallization-driven transitions, or morphologic changes) has been shown using representative examples, including OEGylated polypeptides and glycopolymers, PEG analogs, ionic liquid-based nanogels, methacrylate derivatives, and oxazoline-based polymers. The following observations support the view that solvent selection represents not only a medium choice but also a structural design parameter, which determines sharpness, reversibility, and tunability of thermal phase transitions [44]. The overall significance of the rational selection of solvents and comprehensive knowledge of solvent-specific reactions is emphasized in the collective evidence in the sphere of the development of advanced polymer systems. The equilibrium of the main parameters, which include physicochemical performance, injectability, antimicrobial behavior, and cytocompatibility, is not only needed to optimize in situ forming (ISM) formulations to deliver drugs in a controlled manner but also to design thermo-responsive polymers that can be predictable and tunable. This combined outlook points out that solvent-based molecular interaction is a root determinant of the performance, safety, and functionality of smart materials, biomedical systems, and responsive soft-matter technologies.

### 2.2. Preparation Methods

The method used in synthesis determines the size and shape of the hydrogel, and its functionality, especially when it comes to nano hydrogels [11].

**Bulk Hydrogels**: These are made in large, macroscopic gels, which are less widely used in nanoscale, but provide an intermediate in nano- or micro-sized hydrogel preparation, such as by grinding or emulsification.

**Microgels and Nanogels**: These are hydrogels of particle sizes in the micrometer or nanometer ranges, respectively. Nano hydrogels that are temperature-responsive, like PNIPAM nanogels, are usually prepared using emulsion polymerization or precipitation polymerization, which allows the precise control of the particle size and responsiveness [45].

**In Situ Forming Hydrogels**: These systems are given as liquids that are in situ cured in line with certain stimuli, e.g., temperature. Indicatively, nano hydrogels made of Pluronic or poly(N-isopropylacrylamide) (PNIPAM) exhibit a sol–gel phase transition at physiological temperature, which is why they are very suitable in injectable drug delivery and tissue engineering applications [46].

### 2.3. Biodegradability of Smart Hydrogels

Biodegradability is a critical factor for biomedical applications, particularly for in vivo use [47].

**Biodegradable Hydrogels**: These degrade over time in the body, either through hydrolysis or enzymatic action. Biodegradable temperature-responsive nano hydrogels, such as those based on modified natural polymers, are used for temporary scaffolds or drug delivery systems that do not require surgical removal [48].

**Non-Biodegradable Hydrogels**: These are stable in physiological conditions and are used for long-term applications, such as contact lenses or permanent implants. Synthetic temperature-responsive nano hydrogels, like PNIPAM, are often non-biodegradable unless modified with degradable linkers [49].

Hydrogel classification can be viewed as the basis of comprehending the wide variety of physicochemical characteristics of hydrogels and how to design bioelastic materials in order to apply them to more complex biomedical uses. Of them, temperature-responsive nano hydrogels, which can change dynamically to physiological temperature changes, are one of the most promising categories of materials, and hold promise in the areas of targeted and stimuli-responsive therapeutic systems. Through the characteristics of their sources, composition, structure, cross-linking, sensitivity to stimuli, mode of preparation, and biodegradability, researchers can develop nano hydrogels with optimal characteristics in drug delivery, tissue engineering, biosensing, and others. This flexibility highlights the fact that they can be used to fill gaps in contemporary medicine [50].

## 3. Hydrogel in Nature

### 3.1. Natural Polymers, Copolymers, and Polysaccharides

**Figure 8:**
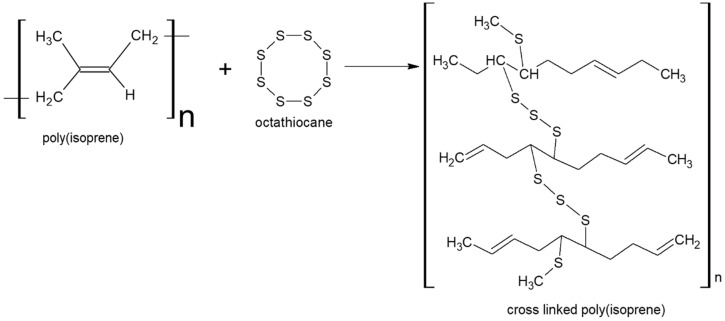
Structural representation of the cross-linking reaction of poly(isoprene), showcasing the formation of a three-dimensional polymer network via covalent linkages between polymer chains.

Polymers are macromolecules that consist of repeated structural elements called monomers, which are small molecules that polymerize into larger molecules [51]. Polymers can be broadly classified into two groups: natural polymers and synthetic polymers. Natural polymers are made of biological or natural origin and can be manufactured by two major processes (i) addition polymerization and (ii) condensation polymerization. Natural polymers include several important biomolecules present in the human body, including proteins or nucleic acids. An outstanding example of a natural polymer is cellulose, which is a major structural constituent of the plant cell walls. It is an organic polysaccharide that consists of long chains of the D-glucose units that are β-linked and give mechanical strength to plants, algae, bacteria, and oomycota. Other examples of polymers found in nature are DNA, silk, wool, pectin, and natural rubber. Specifically, rubber is obtained as latex, a milky liquid derived from rubber trees. Despite being an organic polymer, latex is flattened in a synthesis procedure called vulcanization, in which sulfur cross-links are employed amid chains of poly(isoprene), which changes it into commercial rubber [52]. This is an irreversible chemical reaction between the reactants of poly(isoprene) and octathiocane, as shown in Figure 8. Vulcanization improves the rubber and transforms it from a soft, elastic product to a tough, durable and resistant chemical that can be used in industries and for other commercial purposes.

**Figure 9:**
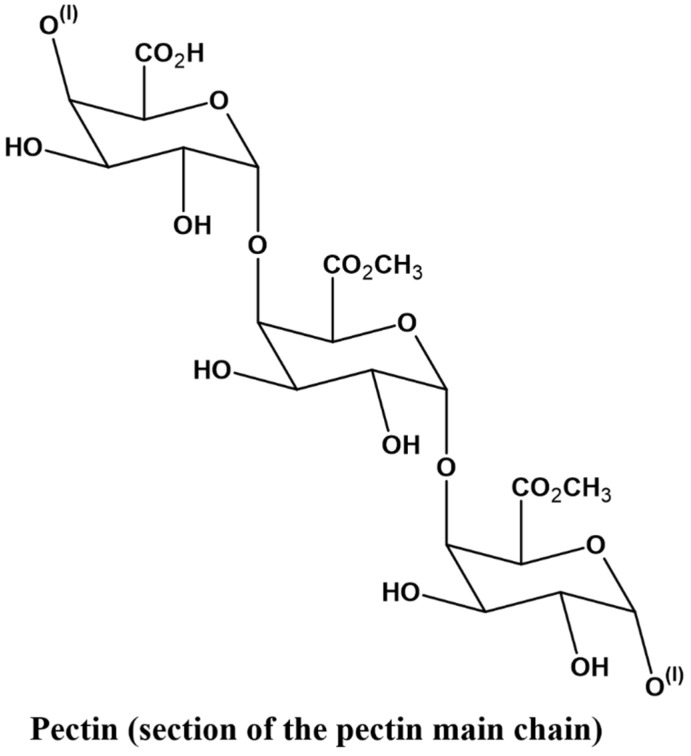
Pectin chains illustrating their linear and branched regions, highlighting galacturonic acid units and potential sites for functional modifications.

Pectin is a long-chain polymer of long chain, consisting mainly of pectin and pectic acid. These acids are formed as a result of sugar molecules; however, pectin is still considered a polysaccharide and is normally extracted from natural sources, which include apple, orange, and lemon peels [53]. It is very critical to the integrity of plant tissues, whereby it connects neighboring plant cells. Figure 9 represents the structural form of the pectin chains.

The pectin chains form a three-dimensional network as they interact with one another through crystallization, developing a structure composed of sugars, water, and other components. When chemical or physical modifications in this network reduce pectin solubility, gelation occurs, enabling the localisation of microcrystals. It shows that the most important process of pectin gel formation is its temperature-sensitive nature. As the heating of a hot pectin solution is reduced, the mobility of the molecules is reduced, and a gel network is formed. Pectin, therefore, serves as a useful thickening agent in foodstuffs like jam, jelly, butter, etc. [54]. To form a stable gel, there must be an adequate concentration of sugars, as is seen in the case of pectin-based gel formation, to show how a thermo-responsive gel can be formed.

**Figure 10:**
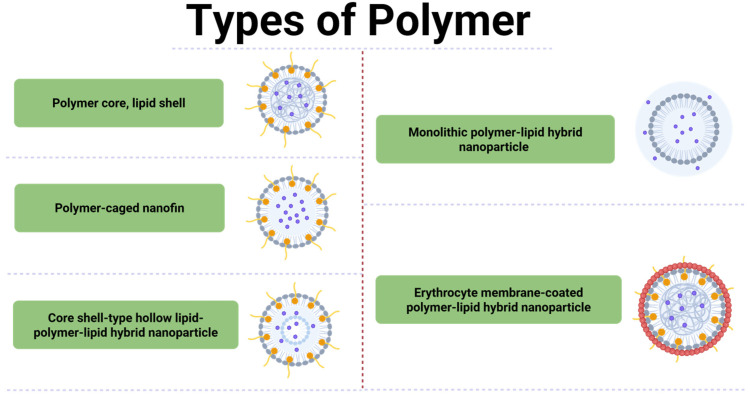
Structural design strategies of polymer–lipid hybrid nanoparticles: schematic representation of different architectures, including polymer core–lipid shell, polymer-caged nanofin, core–shell hollow lipid–polymer–lipid hybrids, monolithic polymer–lipid hybrids, and erythrocyte membrane-coated polymer–lipid hybrids for advanced nanomedical applications.

The production of polymers through the use of a single or several kinds of monomers/biopolymers is known as copolymerization. Terpolymers and quaterpolymers are considered as copolymers with three and four monomers, respectively. Relevant commercially important copolymers are nitrile rubber, styrene acrylonitrile, ethylene vinyl acetate, and butadiene copolymers, which are generally prepared by chain-growth polymerization [55]. The second method of production is the step-growth polymerization, which is used in the production of nylon-6, nylon-6/12, nylon-6/6, and also in polyesters. Depending on the polymer chain arrangement, there exist two types of copolymers: linear and branched (Figure 10). The linear copolymers, those that comprise a single main chain, are the block and statistical copolymers with substitutes. Branched copolymers, conversely, have a single main chain and one or more polymer side chains that can be grafted onto the backbone.

**Figure 11:**
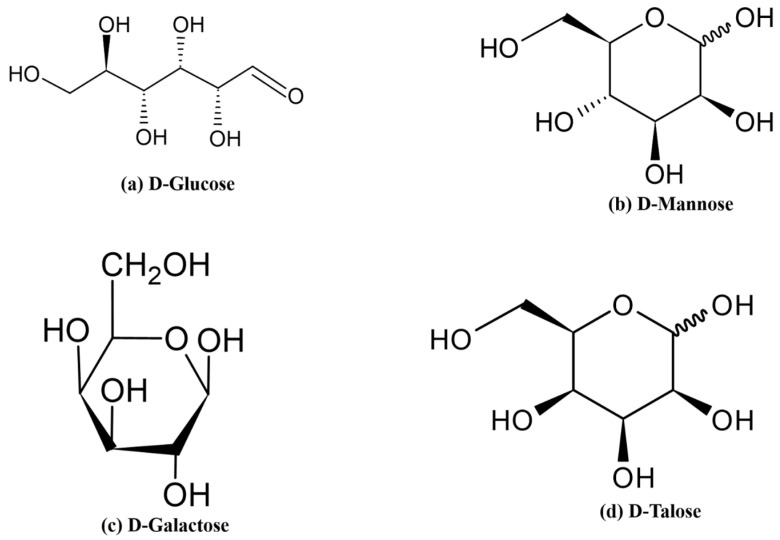
Structural representations of selected polysaccharide monomers, including (**a**) D-Glucose, (**b**) D-Mannose, (**c**) D-Galactose, and (**d**) D-Talose.

In recent decades, considerable studies have been able to prove that a lot of polymers can gel due to temperature variations. Much research has also been conducted on synthetic polymers in which proper combinations of monomers are formed to create thermally sensitive hydrogels with desired characteristics. Polysaccharides (also known as glycans) are polymers that are made up of numerous units of different monosaccharides (Figure 11) [56]. The simple sugars that make up the polysaccharides are referred to as monosaccharides, and they include simple sugars like glucose, which are joined together by glycosidic bonds.

**Figure 12:**
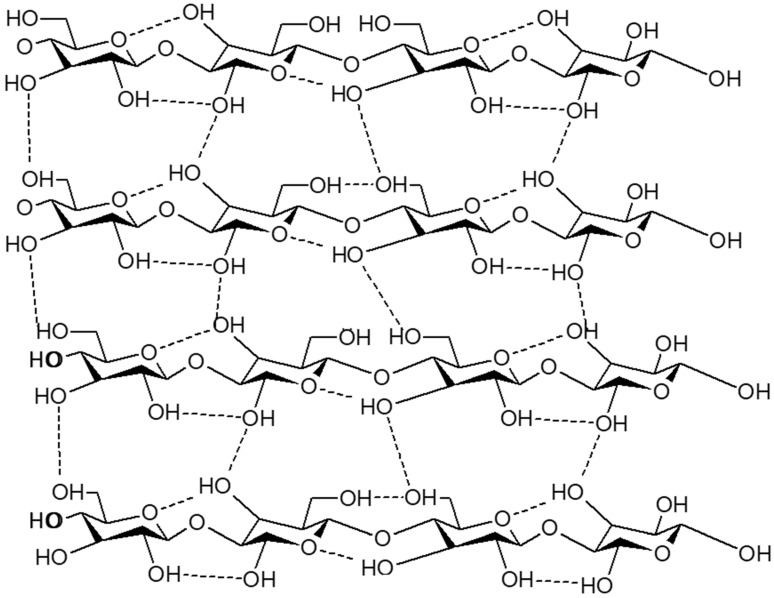
Schematic representation of cross-linked bridged structures in polymer–lipid hybrid nanoparticles, highlighting diverse architectures for enhanced stability, functionality, and biomedical applications.

The nature of the constituent monosaccharides is not the only factor that determines the structural and functional properties of the resulting polysaccharides; the molecules with which the latter interact also affect them. Polysaccharides are energy-storage molecules, which supply the monosaccharides with readily available energy and remain in a tight and stable structure. Monosaccharides can also be used to give structural support, although in chain forms. In such fibrous structures, a large amount of hydrogen bonding among the chains leads to greater mechanical stability of the material [57]. Complex interactions of molecular configurations, side-chain groups, hydroxyl functionalities and enzymatic activity determine the final structure of a polysaccharide. These structures are glycosidic bonds between monosaccharides and these are connected by an oxygen atom connecting two rings of carbon. The glycosidic bond is formed by the loss of one oxygen atom and two hydrogen atoms on the reactants to release a water molecule, which is referred to as a dehydration reaction [58]. The bridged polysaccharide structure obtained is illustrated in Figure 12.

#### 3.1.1. Cellulose Derivatives

Cellulose is a polysaccharide that can be produced naturally and is water-insoluble but can be used to produce thermo-reversible hydrogels [59]. The cellulose solubility can also be improved by replacing its hydroxyl group with a hydrophobic group, e.g., methyl or hydroxypropyl group, which gives it the ability to be soluble in water. Gelation of natural polymers in aqueous solutions can also occur when the solution is cooled, as is seen with carrageenan and gelatin, which both have sol–gel transitions. These polymers are solution-coiled in high temperatures, and when cooled, a partial helix is formed, resulting in a continuous gel network [60]. Some cellulose derivatives, in particular, methylcellulose (MC) and hydroxypropyl methylcellulose (HPMC), have been extensively investigated in terms of their biomedical usage and gelation processes. Cellulose-based hydrogels (e.g., MC/HPMC-K_2_SO_4_) are environmentally responsive and exhibit greater swelling, mechanical stability, and controlled release of fertilizers by Fickian diffusion, which enhances the water retention and growth of plants in soil, and offers a safer alternative to hydrogels using polyacrylamide as the polymer [61]. The aqueous solutions of MC and HPMC are soluble and remain in the liquid state at lower temperatures, even at low concentrations (1–10 wt%). Upon heating, MC loses its solubility in water and undergoes thermo-induced gelation, where the polymer chains swell and associate through hydrophobic interactions. The gelation of HPMC occurs in the range of 75–90 °C, and that of methylcellulose occurs in the range of 40–50 °C. Chemical or physical alterations can be used to modulate these transition temperatures. Spectroscopy, rheology, and differential scanning calorimetry (DSC) are examples of these techniques that can be used to characterize the sol–gel transition of thermo-responsive hydrogels. This gelation is mostly fuelled by the hydrophobic effect, wherein the interaction with water molecules enhances the level of entropy of a system [62]. Examples of structural reorganization that can take place at the critical solution temperature (CST) include coil-to-helix transitions or the formation of polymeric micelles. Natural and synthetic hydrogels such as poly(N-isopropylacryl amide) (pNiPAAm), poly(EOPPO-PEO)-block copolymer, and PEG-based biodegradable polyesters have thermo-reversible gelation in aqueous solution, generally forming gels at 60–80 °C and dissolving on cooling. Zhao et al. grafted MC using synthetic pNiPAAm to form thermo-gelling hydrogels in a fast, reversible way. They were able to control the ratios of the two components, which showed that increased pNiPAAm concentration decreases the lower critical solution temperature (LCST), but increases the LCST and mechanical strength when a higher MC concentration is added [63].

**Figure 13:**
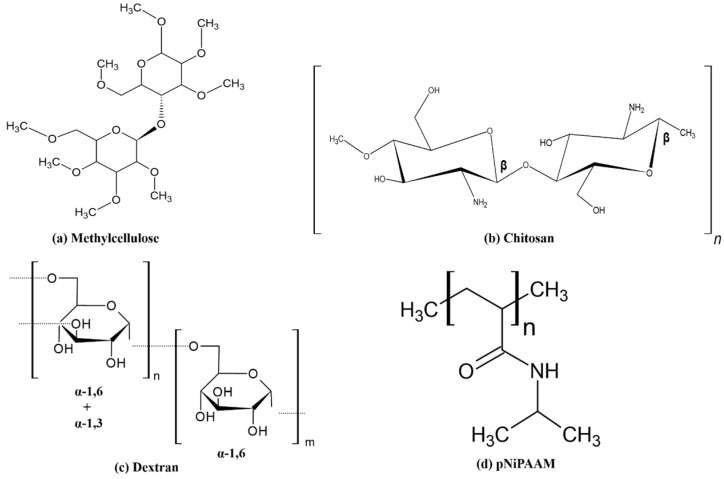
Different types of polymer–lipid hybrid nanoparticles are employed in the biological field, including polymer core–lipid shell, polymer-caged nanofin, core–shell hollow lipid–polymer–lipid hybrids, monolithic polymer–lipid hybrids, and erythrocyte membrane-coated polymer–lipid hybrids.

Klouda et al. have recently reviewed thermosensitive hydrogels made of methylcellulose to be used in biomedical applications, and it can be noted that they have potential in drug delivery and tissue engineering [64,65]. Figure 13 demonstrates some of the elements and types of hydrogels that are usually used in biology.

#### 3.1.2. Chitosan

Yellow thermo-responsible hydrogel made of chitosan has good biodegradability and biocompatibility. But the practicality of some hydrogels may be restricted with respect to their poor mechanical properties. To solve this, a new chitosan/polyol salt-based thermosensitive hydrogel system with physiological pH was designed, which is in the form of a liquid at room temperature but in situ gels at body temperature. This system allows growth factors to be delivered under control, as well as permits encapsulation of the cells, and is therefore very applicable in tissue engineering [66]. Recently, Bhattarai et al. showed that a combination of chitosan and polyethylene glycol (PEG) can provide thermo-reversible hydrogels without the inclusion of other cross-linking reagents [67]. Chitosan is a deacetylated form of chitin and is generally obtained by treating chitin with alkaline substances derived mostly from the shells of crustaceans like crabs. It is a cationic and water-soluble (pH −6.2) polymer and has pH-dependent characteristics and a significant level of biocompatibility. All these properties render chitosan a very versatile compound that can be applied in a wide variety of applications, such as biomedical, agricultural, biopesticides, natural elicitors, biocontrol, filtration, and 3D bioprinting, and as a possible cell carrier in tissue engineering. Moreover, chitosan has thus far been examined as a soluble dietary fiber. The pH-induced gelation of aqueous chitosan solutions causes a hydration-like condition, whereas the pH-induced gelation of aqueous chitosan solutions coupled with the incorporation of polyol salts leads to the formation of thermally sensitive as well as gel-forming hydrogels [68]. Hydrogels that are sensitive to temperature and bioactive, where the gelation temperature is physiological, provide close spatial and temporal regulation and can, therefore, be useful in drug delivery, tissue engineering, imaging, and wound healing [69].

## 4. Methods of Preparation of Hydrogels

Hydrogels can be defined as three-dimensional polymeric networks that are hydrophilic in nature and are usually formed by using mixtures of hydrophilic and hydrophobic monomers to produce specific functional properties of hydrogels, which can be applied to a specific purpose. They are usually made out of natural or artificial polymers. Natural polymer hydrogels are frequently functionalized at the radical group or have natural functional moieties, and synthetic polymer hydrogels are more chemically stable and hydrophobic in nature [70]. Synthetic hydrogel has better mechanical strength and longevity, but it has the drawback of a lower degradation rate.

The issue of balancing these conflicting properties is important and can be attained with the best design approaches. Hydrogels are produced by the establishment of a cross-linked polymeric network, and this gives them elasticity and structural integrity. Hydrogel synthesis is commonly performed by using different cross-linking techniques, such as incorporating multifunctional cross-linkers with hydrophilic monomers through the method of copolymerization [71]. Interconnection using linear, water-soluble synthetic and natural polymers can be performed to create hydrogels by several different mechanisms:

- Physical interactions—crystallization, chain entanglement, and electrostatic interactions.
- Reactions with chemical reactions result in linkages of covalent polymer chains.
- Radicals are induced by ionizing radiation, which form cross-link junctions during the recombination.

These can be applied in solutions, suspensions, or bulk polymerization. The major requirements of hydrogel formation are monomers, initiators, and cross-linkers, and the presence of water or other aqueous solutions is usually used as a diluent to control the kinetics and properties of hydrogelation. Hydrogels are washed after synthesis to eliminate any unreacted monomers, cross-linkers, initiators, and by-products that result during side reactions.

**Figure 14:**
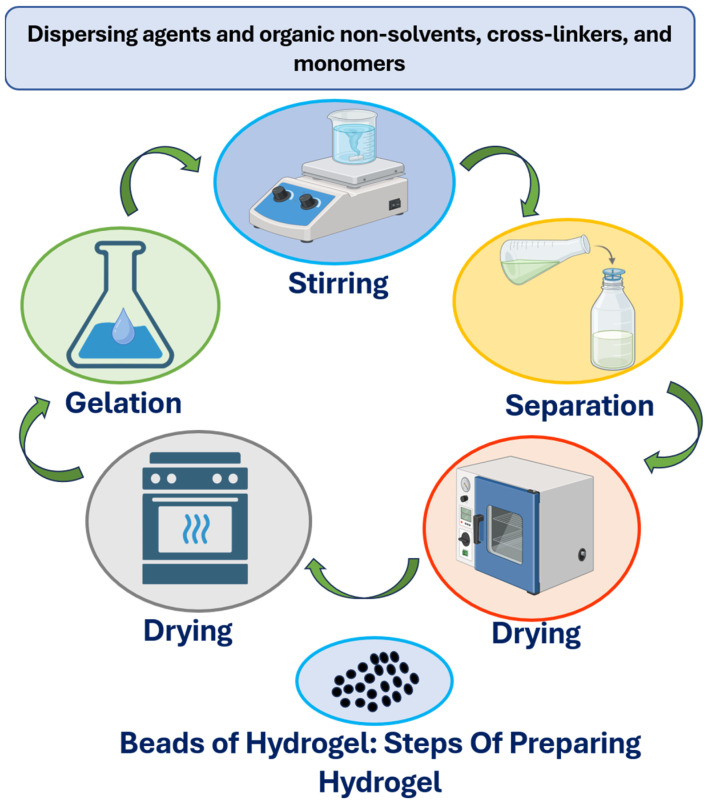
Sequential process for hydrogel bead preparation involving stirring, separation, gelation, and drying with dispersing agents, cross-linkers, and monomers.

Acrylic monomers and their salts are widely studied as hydrogels, most of which are diluted solution polymerization, inverse suspension polymerization, and highly purified solution polymerizations, which are widely used and many of which are patented. As an example, Chen described the preparation of a superabsorbent hydrogel of acrylic acid–sodium acrylate (43.6 wt%) in the presence of potassium persulfate as a thermal initiator [72]. The manufacturing of a cross-linked polymer network that forms a hydrogel uses various methods. Radiation cross-linking is a technique that incorporates high-energy radiation in order to create covalent bonds between polymer molecules, forming mechanically stable hydrogels. Physical cross-linking, in contrast, is based on non-covalent interactions, e.g., hydrogen bonds or ionic interactions, to create reversible networks. Graft polymerization involves attaching polymer chains to a backbone, enhancing network stability and functionality. Chemical cross-linking, achieved through covalent bond formation using cross-linking agents, provides precise control over the hydrogel’s structure and properties. These fabrication methods enable specific adjustment of the hydrogel’s viscoelasticity and mechanical characteristics, which increases its applicability in a vast array of pharmaceutical and biomedical applications, such as drug delivery systems, tissue-engineering scaffolds, and wound dressing [73]. The schematic view on the formation of hydrogel beads is shown in Figure 14. Broadly speaking, the techniques of hydrogel preparation are as follows:

### 4.1. Chemical Cross-Linking Method

Chemical cross-linking involves using covalent bonds in order to cross-link polymer chains, creating a network that is three-dimensional. Such a construction offers increased mechanical strength, barriers and water-resistance, but the mobility of the polymer chains is greatly lowered, and there are variations in the physicochemical properties of the polymer [74]. Such networks are formed through reactions of functional groups (e.g.,-COOH, -NH2, -OH) present on the polymers with cross-linking agents like aldehydes (e.g., glutaraldehyde, adipic acid dihydrazide). Such reactions typically entail attaching monomers to the polymer backbone and allowing two chains of polymers to be covalently attached. In the literature, there are multiple ways of attaining chemically permanent cross-linked hydrogels. One of the methods is hydrophobic interaction, which is used when polar hydrophilic groups are oxidized or hydrolyzed to engage in covalent cross-linking. The other method is the use of interpenetrating polymer networks (IPNs), whereby a monomer is polymerized inside an existing solid polymer network to form an intertwining network, and thus, mechanically stable hydrogels are obtained [75]. Popular chemical cross-linking techniques to be applied to make hydrogels of natural polymers are as follows:

- Grafting techniques
- Cross-linking by radiation (aqueous or solid)
- Chemical cross-linkers

The methods allow creating hydrogels with custom mechanical, chemical, and functional characteristics that can be used in a variety of biomedical and industrial applications.

#### 4.1.1. Grafting Method

**Figure 15:**
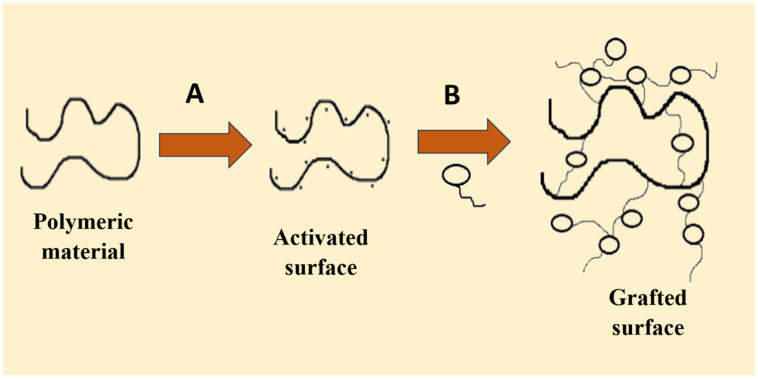
Grafting method: polymeric backbone formation leading to branching and cross-linkage; A = gamma rays, plasma treatment, chemical treatment, electron beam exposure, and B = functional monomer at inert atmosphere.

The polymerization of the different monomers on the polymer backbone occurs in this process. Activation of the polymer chains is achieved using either high-energy radiation or a chemical reagent. When this is triggered, the monomer units proceed to propagate via the macroradicals, which also enhance further branching and create a cross-linked network structure [76]. The grafting can be conducted in by either chemical grafting or radiation grafting, like in Figure 15.

#### 4.1.2. Radiation Cross-Linking Method

**Figure 16:**
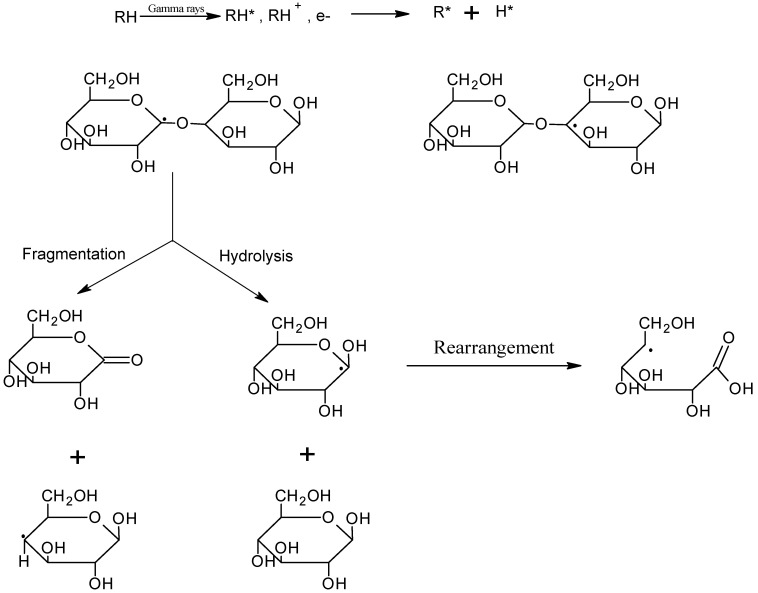
Solid-state radiation effects on hydrocolloids, illustrating structural modifications, cross-linking, and physicochemical changes induced by irradiation.

The benefits of radiation grafting are that no extra chemical additives are required; hence, the biopolymer is not lost in terms of biocompatibility. It is a relatively inexpensive method of modifying and sterilizing biopolymers, and it is, therefore, very appropriate in the biomedical world. The process is based on the free radical production of the polymer chains and on taking advantage of the high-energy sources like electron beams, X-rays, or gamma radiation [77]. The efficacy of radiation-induced modifications depends on the physical conditions and concentration of the polymer, be it in concentrated solution, solid form, or in a dilute solution, and on the energy transfer mode, which may be either direct or indirect. Radiation cross-linking can be subdivided into aqueous-state, paste, and solid-state radiation, and solid radiation is further subdivided into the cross-linking of natural polymers or cross-linking of synthetic and natural polymers. Figure 16 shows the mechanism of the reaction that takes place in solid-state radiation.

### 4.2. Physical Cross-Linking Method

The last few years have seen increasing attention paid to physically prepared hydrogels because of the simplicity of their production, which does not presuppose the use of chemical cross-linking agents. This is mainly beneficial in maintaining the structural and functional integrity of the entrapment of substances like proteins or cells, which may be negatively influenced by chemical cross-linkers. The gelation of hydrogels through physical cross-linking is also highly controllable by fine-tuning the various parameters: polymer concentration, pH, and selection of the types of hydrocolloids, allowing a large variety of gel textures and mechanical strengths [39]. The physical cross-linking technique is also becoming popular, particularly in the food industry. The literature has reported many techniques, such as

- Polymer solutions: thermal treatment (cooling or heating) of polymer solutions
- Cooling/heating a polymeric solution
- Ionic interaction
- Hydrogen bonding
- Aggregation (maturation) by heat.
- Freeze–thawing

These methods offer a wide range of methods for obtaining hydrogels with desirable functional and structural properties without utilizing chemical cross-linkers.

#### 4.2.1. Encapsulation Through Gelatin-Based Chemical Approaches

Encapsulation with gelatin is based on chemical techniques to create polymeric droplets by the suspension technique, in which a suspension technique is used to separate two liquid phases that are immiscible in a concentrated colloidal system. During this, the formation of gels is through the interaction of the polycations and polyanions [78]. The rationale is that the opposite polarly charged polymers combine to create a soluble or insoluble complex; its quality will rely on the pH and the level of the respective solvents.

#### 4.2.2. Ionic Interaction

Here, ionic polymers are cross-linked with the help of specific interactions with divalent or trivalent counterions [79]. The technique relies on the polyelectrolytic solution gelling concept, e.g., Na^+^ and Ca^2+^ or Na^+^ and Cl^-^ interplay. Examples of them are chitosan–dextran hydrogels and chitosan–glycerol phosphate salt hydrogels.

#### 4.2.3. Freeze–Thawing

Freeze–thawing is a process in which polymer solutions undergo repeated freezing and thawing treatments, which cause the physical cross-linking of the solution and formation of a hydrogel. These cycles favor the development of microcrystalline structures in the network. Freeze–thaw hydrogels such as xanthan and polyvinyl alcohol (PVA) gels are examples [80].

## 5. Applications of Hydrogels

Nano hydrogels with temperature responsiveness, including those derived using PNIPAM, Pluronic and PEG-PPO copolymers, have reversible phase changes at physiological temperatures, allowing them to be highly tuned to biomedical uses. Their tailorable designs enable controlled drug delivery, injectable tissue-engineering scaffolds, and sensitive biosensing systems as well as adaptive wound-healing systems that react to body temperature.

### 5.1. Biomedical Applications of Hydrogels

The swelling capacity, porosity, and water-retention attributes of hydrogels have made their use widespread in the biomedical sector [81]. Other applications outside the biomedical field include food, applications in the environmental sector, and in other industries [82]. Since the human body is 60–75% water, the capacity to absorb and retain water of hydrogels makes them especially applicable in the biomedical sphere [83]. The research has been conducted in recent decades on the use of hydrogels in cell culture, tissue engineering, self-healing materials, hemostatic bandages, biosensors, and drug delivery systems [84,85,86]. Hydrogels are tunably biodegradable, porous, mechanically tough, and biocompatible. Although natural hydrogels have the benefit of being biocompatible, synthetic hydrogels are commonly used due to their increased strengths and durability in gels, together with water absorption. These properties may be designed with certain compositions, with all-synthetic or hybrid polymeric systems. Synthetic hydrogels can also be designed to be stable at extreme conditions (high or low pH, high temperature) or to respond to external stimuli, such as chemical agents, magnetic fields, heat, pH, light, or functional groups [87]. It has been applied in self-healing materials, three-dimensional cell-culture matrices, drug delivery systems, tissue-engineering scaffolds, contact lenses, and hygiene products.

#### 5.1.1. Hydrogels in Self-Healing

Self-healing is the spontaneous reconstruction of new connections after a break in the existing connections, similar to the natural healing process that is observed in wood, skin, and bone [88]. This is usually not the case with conventional synthetic hydrogels, and efforts are underway to develop materials with better mechanical characteristics and self-healing properties. Hydrogels may also be covalently or non-covalently self-healing. The biological healing process usually depends on the theory of energy dissipation, which involves breaking and reforming sacrificial bonds as a reaction to stress. New advances are hydrogel-based hydrogels that have reversible oxime cross-links, thus allowing autonomous healing [89]. They are produced by means of diacetone acrylamide (DAA) and keto-functional monomers, which are co-polymerized with the help of a free radical with N,N-dimethylacrylamide (DMA). Further additions of monofunctional alkoxyamines make it possible to have reversible gel–sol transitions at ambient temperatures [90]. Also, Okay and colleagues described the formation of hydrogel through the copolymerization of hydrophobic acrylate monomers (dodecyl acrylate, stearyl methacrylate) and hydrophilic acrylamide in micellar solutions of sodium dodecyl sulfate, which showed that the properties of the hydrogel could be tuned by the choice of monomers and the polymerization conditions. Abeer and co-workers also reported the optimized thermosensitive in situ gel of Latanoprost which demonstrated superior ocular retention, enhanced permeability, and sustained anti-glaucoma efficacy compared to conventional eye drops. Its favorable stability and prolonged IOP reduction suggests it as a promising non-invasive alternative for effective glaucoma management [91].

#### 5.1.2. Hydrogels in 3D Cell Culture and Tissue Engineering

Earlier research has stressed that tissues need both proper mechanical characteristics as well as elevated water contents to promote cellular accumulation and the development of more intricate tissue structures in vivo and in vitro. An artificial engineered environment that recreates the natural conditions well will be required to produce three-dimensional (3D) regenerative cell constructs with high accuracy, which stimulates cell growth and spontaneous interactions in all three dimensions. Anseth and co-workers presented a new cross-linked chemistry based on tetrazine–norbornene click reactions to produce cell-laden hydrogels that can be used in cell culture in 3D. The reaction kinetics, bio-orthogonality, and photochemical compatibility of the hydrogel make it extremely applicable in translational tissue engineering as well as basic biological research [92].

Roshanbinfar et al. produced a GA-functionalized hyaluronic acid (HA)/collagen I/multi-walled carbon nanotube (MWCNT) composite hydrogel to be used in cell culture. This hydrogel exhibits higher mechanical stability, antioxidative properties, and shape fidelity, which is effective in reducing the shrinkage of the matrix. Its high level of biocompatibility and printability makes it a promising scaffold for tissue engineering and organ-on-chip systems [93].

#### 5.1.3. Hydrogels in Drug Delivery

Hydrogel is commonly used in drug delivery systems, whereby hydrogel is synthesized ex vivo and loaded with a therapeutic agent and the ex vivo gel is delivered to the body. The cross-linking techniques used in different studies are various and they include UV polymerization and chemical cross-linking reactions [94,95]. Hydrogels are extensively used in biomedical research, experimental medicine, and clinical practice (cell immobilization, control of biological adhesion, barrier materials, diagnostics, separation of cells or biological molecules, regenerative medicine, and tissue engineering [96].

A porous hydrogel matrix is necessary to deliver drugs effectively because it shields the drug against adverse environmental factors and is used to load the drug. The porosity of the hydrogel is determined by the cross-linking density of the hydrogel, whereas the rate of drug release is mainly determined by the diffusion coefficient of the drug molecules within the hydrogel structure [97]. The biodegradability and biocompatibility of the hydrogel can be achieved through the design of the chemical and physical structure to improve its use as a drug delivery platform. Poloxamer (pluronic) is a triblock copolymer consisting of poly(ethylene oxide)-b-poly(propylene oxide)-b-poly(ethylene oxide) (PEO-PPO-PEO) and is one of the widely used drug materials in pharmaceuticals. Paavola and colleagues designed a hydrogel system using Poloxamers, which is injected to release the anesthetic drug lidocaine in a controlled and reproducible manner, thus proving its usefulness in commercial and clinical applications. The kinetics of drug release in the hydrogel can also be controlled with functional groups, such as carbohydrates, amines, and ethoxysilanes, which stabilize the polymer network and slow down fast dilution in the aqueous environment.

Wu and co-workers demonstrated that peptide-based supramolecular hydrogels represent a multipurpose and effective platform for targeted and responsive biomedical therapies, enhancing drug efficacy while minimizing side effects. Continued research addressing current challenges will further expand their potential for personalized and advanced disease treatments [98]. Xiong developed a novel injectable doxorubicin-loaded hydrogel, formed via host–guest inclusion of adamantane–doxorubicin into β-cyclodextrin-modified polyaldehyde dextran and in situ cross-linking with carboxymethyl chitosan [99,100].

Smart hydrogels are becoming the future biomaterials of choice to be used as a localized therapy in complex and moist structures like the vaginal canal area and periodontal pockets. Their ability to adhere to mucosal surfaces, maintain drug release, and respond to pathological signals (e.g., pH, reactive oxygen species or microbial enzymatic activity) enables them to address the key limitations of traditional topical or systemic delivery. The advanced periodontal applications, as evidenced, show that injectable, adhesive, and stimuli-responsive hydrogels are more than capable of targeting therapeutic agents, removing pathogenic biofilms, regulating inflammation, and regenerating tissue under dynamic intraoral fluid flow conditions [101]. The same material properties are directly applied to the vaginal drug-delivery requirements, where the capability to retain the drugs over extended periods, protect labile drugs, and control release in response to microenvironment changes is essential in the treatment of infections, tissue remodeling, and the delivery of anti-inflammatory or antimicrobial agents. Their capacity to experience sol–gel transitions in vivo, attach to moist tissues and deliver sustained and stimulus-regulated release allows them to circumvent the short half-life of drugs that follows physiologic fluid flow, enzyme activity, and microbial loading. Experiments using periodontal-based applications support the use of stimuli-responsive hydrogels such as thermo-, pH-, ROS-, light-, and enzyme-responsive hydrogels to precisely target diseased pockets, inhibit pathogenic biofilms, regulate inflammatory reactions, and stimulate tissue regeneration even in the in vivo conditions of chewing and salivary flow [102].

Vaginal infections still remain a major health issue for women because of high rates of recurrence, growing resistance to antimicrobials, and the lack of elimination of pathogenic strains. Although conventional vaginal preparations are available, there is difficulty in improving their therapeutic effect due to the special anatomy and physiology of the vaginal environment, especially the fluid secretion and the renewal of the mucosa, which makes the vagina able to rapidly clear the administered drugs. Hydrogels are potential systems to target vaginal therapy in a localized way, with long residence time in the location of action and the ability to adjust drug-release characteristics. The addition of certain polymers can also provide these systems with mucoadhesive, stimuli-responsive, or intrinsic antimicrobial characteristics, thus increasing their affinity to vaginal mucosa and therapeutic efficacy. This review identifies current developments, strengths, and weaknesses of hydrogels in delivering drugs or nanocarriers as one of the systems of controlling vaginal infections, with particular focus on polymers and functional characteristics most commonly utilized to maximize the treatment. It also offers knowledge that can be used to make better localized treatments of bacterial vaginosis, vulvovaginal candidiasis, and trichomoniasis [103]. The introduction of smart hydrogel systems into vaginal and periodontal therapies is a major step towards specific, local and patient-adherent drug delivery platforms that can help to improve therapeutic outcomes in difficult mucosal conditions.

#### 5.1.4. Hydrogels in Tissue Engineering

**Figure 17:**
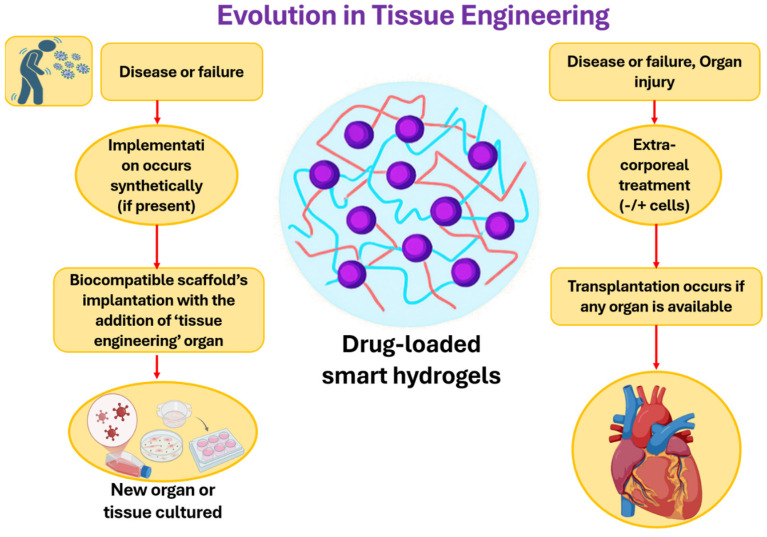
Comparison of conventional transplantation and advanced tissue engineering using drug-loaded smart hydrogels for organ and tissue regeneration.

Hydrogels have been highly studied in tissue engineering to meet the high demand for functional tissues and organs [104]. The objective of tissue engineering is to produce and substitute damaged or absent biological structures (Figure 17) [105], owing to the inexhaustible scarcity and increasing discrepancy in organ and tissue transplantation. Hydrogels are critical in such an area and serve as biocompatible and water-swollen three-dimensional scaffolds that mimic the extracellular matrix, therefore, promoting cell adhesion, proliferation, and tissue development. With only 23,407 transplants performed from July 2000 to 2025 against a waiting list of 79,902, hydrogels address this gap by supporting tissue regeneration for applications like cartilage, bone, and skin. Available as natural, synthetic, or hybrid forms, they offer tunable properties but face challenges in mechanical strength, vascularization, and scalability. Advances like 3D bioprinting and smart hydrogels are enhancing their potential to revolutionize organ replacement [106,107].

### 5.2. Applications in Other Fields

Since the pioneering development of synthetic hydrogels by Lim and Wichterle in 1960 for biomedical applications, these water-swollen, three-dimensional polymer networks have undergone significant advancements, expanding their utility across multiple disciplines [108]. Temperature-responsive hydrogels, which exhibit reversible sol–gel phase transitions in response to minor temperature fluctuations, have emerged as highly adaptable platforms. Their tunable physicochemical properties and responsiveness to external stimuli have enabled transformative applications in agriculture, industrial processes, food technology, hygiene products, diagnostics, biotechnology, and biomedicine [109]. This section reviews the diverse applications of hydrogels, highlighting their versatility and impact beyond their initial biomedical scope [110].

In agriculture, these hydrogels function as efficient soil conditioners and water-retention agents, enhancing crop yields and optimizing water use in arid and semi-arid regions [111,112]. Their environmentally friendly and non-toxic properties also make them ideal for producing artificial snow, providing a sustainable alternative for recreational and sporting activities. In the food industry, temperature-responsive hydrogels serve as stabilizers, thickeners, and controlled-release systems for additives and flavorings, improving product shelf life and sensory qualities. Additionally, their swelling properties enable effective sealing and moisture-resistant packaging solutions [113].

In the hygiene sector, superabsorbent hydrogel-based polymers are essential in products such as diapers, sanitary napkins, and adult incontinence pads, offering superior absorbency and user comfort [114]. In diagnostics, hydrogels enhance the sensitivity of immunoassays and lab-on-chip devices through their porous and responsive structures, enabling precise biomarker detection [115]. Industrially, these materials are utilized in coal dewatering and wastewater treatment, leveraging their capacity to absorb and immobilize large volumes of water for efficient solid–liquid separation. In biotechnology, their tunable pore structures and selective binding properties facilitate the separation and purification of cells, proteins, and biomolecules.

**Figure 18:**
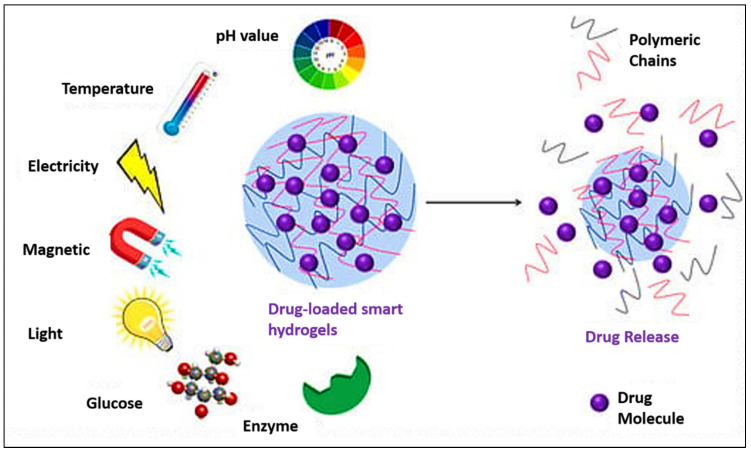
Schematic representation of stimuli-responsive drug-loaded smart hydrogels. External stimuli such as pH, temperature, electricity, magnetic field, light, glucose, and enzymes trigger structural changes in the hydrogel network, leading to polymer chain rearrangement and controlled drug release of encapsulated therapeutic molecules. Reprinted with Creative Commons CC BY 4.0 [118].

The temperature-responsive hydrogel has found significant use in biomedical practice, like wound dressings. They provide a moist healing environment, promote tissue regeneration, and permit the controlled introduction of antimicrobial or therapeutic agents, which positively affect overall wound care effectiveness [116]. Their dynamic swelling/deswelling behavior also makes them ideal for biosensors, enhancing sensitivity and response time to physiological stimuli. Furthermore, hydrogels are increasingly used to regulate biological barriers, mimicking or reinforcing epithelial and endothelial tissues, which holds significant promise for tissue engineering and regenerative medicine [117] (Figure 18).

The multifunctionality of temperature-responsive hydrogels highlights their pivotal role across scientific, industrial, and biomedical domains [119]. As innovations in hydrogel chemistry and nanocomposite design continue to advance, these smart materials are poised to drive the development of next-generation solutions, addressing critical challenges in healthcare [120], environmental sustainability [40], and beyond.

## 6. Future Scope

Although hydrogels may be classified according to their source, polymeric composition, structural arrangement and cross-linking mechanisms, these core characteristics essentially determine their ability to exhibit a wide range of stimuli-responsive behaviors. The intrinsic biocompatibility of natural polymers, the mechanical tunability of synthetic matrices, and the multifunctional synergy of hybrid systems all affect the response of hydrogels to external stimuli, such as temperature, pH, ionic strength, light, redox conditions, or magnetic fields. In addition, molecular structures, whether homopolymeric, copolymeric, or IPN-based, predetermine the accuracy, acuity, and reversibility of such transitions, allowing for the control of swelling, sol–gel dynamics, and the kinetics of drug release with high precision. Structural stability and adaptability are further regulated by the choice of physical, chemical or dual cross-linking, which dictates the appropriateness of hydrogels in dynamic conditions. The knowledge of these basic classifications forms a fundamental basis that underpins the next section, where we narrow down the stimuli-responsive classification of hydrogel, in which external stimulus influences the functional changes in hydrogel in biomedical, environmental, and industrial use. In reference to this, the manuscript also briefly mentions smart hydrogel systems in the future outlook, considering their increased significance in the future of materials science. Smart hydrogels, with the ability to respond to multiple stimuli, autonomous flexibility, self-healing, and programmable, are a positive step towards more advanced diagnostics, regenerative medicine, targeted drug delivery, soft robotics, and environmental sensing. The combination of nanotechnology, AI-driven design, and bioinspired engineering presents enormous perspectives of future research. In this way, the stimuli-responsive classification, which is presented here and which has been at the heart of present-day hydrogel technologies, will not only be the foundation of the further advancement of technologies of more advanced smart hydrogel systems in the future but it will also precondition the latter.

## 7. Conclusions

Temperature-sensitive hydrogels have become one of the foundations of research on smart biomaterials, where polymer chemistry and biomedical innovation meet. Their special capability of reversible sol–gel transitions at physiological temperatures has facilitated advances in controlled drug delivery, tissue regeneration, 3D cell culture, wound healing, and biosensing. This review has indicated that their structural design, composition, and cross-linking strategies, be it physical, chemical, or hybrid, are important factors that dictate their swelling behavior, mechanical stability, and biocompatibility. Further improvement in their performance has been achieved by incorporating natural polymers, such as chitosan and cellulose derivatives, into synthetic systems, such as PNIPAAm and Pluronics, which enable fine-tuning of responsiveness and degradability for clinical use. However, despite significant progress, there are still a number of obstacles to the translation of temperature-responsive hydrogels from the laboratory to clinical practice. There are still problems of low mechanical strength, low response time, cytocompatibility in physiological conditions, and reproducibility on a large scale. Furthermore, accurate control over the temperature of phase transition and drug release kinetics is required in order to guarantee therapeutic efficacy and safety.

The next generation of temperature-responsive hydrogels will likely be the brainchild of nanotechnology, computational modeling, and artificial intelligence-driven design. The ability to create intelligent hybrid hydrogels that are responsive to various stimuli, e.g., pH, magnetic field, or light, will open new opportunities of on-demand and site-specific therapies. Combined with bioelectronics and soft robotics, injectable, shape-memory, or self-healing technologies can be used to provide real-time physiological monitoring and adaptive delivery of treatment. Moreover, green chemistry and biodegradable precursors will be used in sustainable synthesis methods that will promote environmentally friendly production and clinical safety. The future of regenerative medicine will be interdisciplinary research in materials science, biotechnology, and medical engineering to produce personalized, multifunctional hydrogels that recapitulate natural tissues, promote organ regeneration, and transform regenerative medicine. Altogether, temperature-responsive hydrogels are on the edge of the new generation of biomedical materials, which will revolutionize the healthcare sector due to intelligent design, adjustable performance, and sustainable innovation.

## Data Availability

No new data were created or analyzed in this study. Data sharing is not applicable to this article.

## Abbreviations

The following abbreviations are used in this manuscript:

Abbreviation	Full Form
3D	Three-Dimensional
AI	Artificial Intelligence
CST	Critical Solution Temperature
DSC	Differential Scanning Calorimetry
ECM	Extracellular Matrix
FTIR	Fourier-Transform Infrared Spectroscopy
HA	Hyaluronic Acid
HPMC	Hydroxypropyl Methylcellulose
IPN	Interpenetrating Polymer Network
LCST	Lower Critical Solution Temperature
MC	Methylcellulose
MWCNT	Multi-Walled Carbon Nanotube
NIR	Near-Infrared
NMP	N-Methyl-2-Pyrrolidone
NMR	Nuclear Magnetic Resonance
PEO	Poly(ethylene oxide)
PEG	Polyethylene Glycol
PG	Propylene Glycol
PNIPAM/PNIPAAm	Poly(N-isopropylacrylamide)
PVA	Polyvinyl Alcohol
ROS	Reactive Oxygen Species
SEM	Scanning Electron Microscopy
TEM	Transmission Electron Microscopy
UCST	Upper Critical Solution Temperature
